# Health Care, Family, and Community Factors Associated with Mental, Behavioral, and Developmental Disorders and Poverty Among Children Aged 2–8 Years — United States, 2016

**DOI:** 10.15585/mmwr.mm6750a1

**Published:** 2018-12-21

**Authors:** Robyn A. Cree, Rebecca H. Bitsko, Lara R. Robinson, Joseph R. Holbrook, Melissa L. Danielson, Camille Smith, Jennifer W. Kaminski, Mary Kay Kenney, Georgina Peacock

**Affiliations:** ^1^Epidemic Intelligence Service, CDC; ^2^Division of Human Development and Disability, National Center on Birth Defects and Developmental Disabilities, CDC; ^3^Division of Congenital and Developmental Disorders, National Center on Birth Defects and Developmental Disabilities, CDC; ^4^Office of Epidemiology and Research, Maternal and Child Health Bureau, Health Resources and Services Administration, Rockville, Maryland.

## Abstract

Childhood mental, behavioral, and developmental disorders (MBDDs) are associated with adverse outcomes that can persist into adulthood (*1*,*2*). Pediatric clinical settings are important for identifying and treating MBDDs (*3*). Early identification and treatment of MBDDs can promote healthy development for all children (*4*), especially those living in poverty who are at increased risk for MBDDs (*3*,*5*) but might have reduced access to care (*6*). CDC analyzed data from the 2016 National Survey of Children’s Health (NSCH) on MBDDs, risk factors, and use of federal assistance programs (e.g., Supplemental Nutrition Assistance Program [SNAP]) to identify points to reach children in poverty. In line with previous research (*3*,*6*), compared with children in higher-income households, those in lower-income households more often had ever received a diagnosis of an MBDD (22.1% versus 13.9%), and less often had seen a health care provider in the previous year (80.4% versus 93.8%). Among children living below 200% of the federal poverty level (FPL) who did not see a health care provider in the previous year, seven of 10 were in families receiving at least one public assistance benefit. Public assistance programs might offer collaboration opportunities to provide families living in poverty with information, co-located screening programs or services, or connection to care.

NSCH is a national, cross-sectional, web-based and paper-based survey funded and directed by the Health Resources and Services Administration’s Maternal and Child Health Bureau that is representative of noninstitutionalized children aged 0–17 years in the United States.* The U.S. Census Bureau conducted the 2016 NSCH using address-based sampling and created weights to account for oversampling and potential nonresponse biases.^†^ Parents were asked, “Has a doctor or other health care provider ever told you that this child has (specified MBDDs)?” A child was considered to have ever had an MBDD if their parent reported one or more of the following: anxiety problems, depression, attention-deficit/hyperactivity disorder, behavioral or conduct problems, Tourette syndrome, autism spectrum disorder, learning disability, intellectual disability, developmental delay, or language problems. Parents also responded to questions related to factors associated with MBDDs (*1*,*3*), including household income, health insurance, components of a medical home, difficulty getting by on the family’s income, parent emotional support, neighborhood condition (e.g., litter or vandalism), neighborhood amenities (e.g., sidewalks or parks), and parental mental or physical health, as well as whether they received public assistance (e.g., SNAP; Women, Infants, and Children [WIC]; free or reduced price meals at school; or cash assistance).^§^

Parents of 50,212 children participated in the survey, resulting in an interview completion rate of 69.7% and a weighted response rate of 40.7%. Analyses were restricted to children aged 2–8 years with nonmissing data on MBDD diagnosis and age (16,912 children). Data missing on race (0.3%), ethnicity (0.5%), sex (0.1%), and FPL (16.6%) were imputed using hot-deck imputation (a method for handling missing data in which missing values are replaced with observed responses from “similar” units) and regression methods.^¶^ Differences in demographic, health care, family, and community factors by MBDD status were assessed using weighted prevalence estimates, prevalence ratios (PRs), 95% confidence intervals (CIs), and Wald chi-square tests. Prevalence of MBDDs, health care, family, and community factors were compared by FPL category. Weighted prevalence estimates, PRs, and 95% CIs were calculated. To further explore whether federal assistance programs are possible points to reach children living in poverty, 4,410 children living below 200% of the FPL who had and had not seen a health care provider in the past year, both with and without MBDDs, were compared by whether their families received public assistance. Statistical software was used to account for the complex survey design.

Overall, 17.4% of children aged 2–8 years had at least one MBDD (Table 1). Child sex, age, and race/ethnicity varied by MBDD status. Compared with children without MBDDs, those with MBDDs more often lived in the lowest income category (<100% of FPL; PR = 1.4) and less often in the highest category (≥400% of FPL; PR = 0.8). Prevalences of most risk factors (e.g., child care problems, and lack of support in neighborhood) were higher among children with MBDDs than among those without MBDDs.

**TABLE 1:** Prevalence of demographic, health care, family, and community factors, by parental report of any mental, behavioral, or developmental disorder (MBDD)* among children aged 2–8 years — National Survey of Children’s Health, United States, 2016

Characteristic	Any MBDD	No MBDD	Any MBDD/No MBDD prevalence ratio (95% CI)	p-value^§^
	% (95% CI)^†^	% (95% CI)^†^		
**Overall**	17.4 (16.2–18.7)	82.6 (81.3–83.8)	—	—
**Child sex**				
Male^¶^	66.7 (63.0–70.1)	47.8 (46.0–49.6)	1.4 (1.3–1.5)	<0.001^§^
**Child age group (yrs)**				
2–3	18.0 (15.1–21.3)	30.4 (28.9–32.0)	0.6 (0.5–0.7)	<0.001^§^
4–5	25.0 (21.7–28.5)	29.2 (27.6–30.9)	0.9 (0.7–1.0)	0.028^§^
6–8	57.0 (53.1–60.8)	40.4 (38.5–42.2)	1.4 (1.3–1.5)	<0.001^§^
Child race/ethnicity**				
White, non-Hispanic	53.6 (49.6–57.5)	51.7 (49.9–53.6)	1.0 (1.0–1.1)	0.405
Black, non-Hispanic	13.8 (11.2–16.9)	11.5 (10.3–12.8)	1.2 (1.0–1.5)	0.137
Hispanic	24.2 (20.1–28.7)	24.4 (22.4–26.5)	1.0 (0.8–1.2)	0.940
Other, non-Hispanic	8.4 (7.1–10.0)	12.4 (11.5–13.5)	0.7 (0.6–0.8)	<0.001^§^
**Parent education**				
Less than high school	8.7 (6.0–12.4)	7.7 (6.2–9.5)	1.1 (0.7–1.7)	0.577
High school	19.9 (16.7–23.6)	17.2 (15.6–18.8)	1.2 (1.0–1.4)	0.154
More than high school	71.4 (67.1–75.3)	75.2 (73.1–77.1)	0.9 (0.9–1.0)	0.107
**Language**				
Primary language other than English	11.0 (7.8–15.4)	15.5 (13.7–17.4)	0.7 (0.5–1.0)	0.035^§^
**Urban/Rural designations^††^**				
Urban	89.6 (87.6–91.3)	91.1 (90.4–91.8)	1.0 (1.0–1.0)	0.136
Large rural	6.2 (4.8–8.0)	5.1 (4.6–5.7)	1.2 (0.9–1.6)	0.198
Small rural	2.6 (1.9–3.5)	2.2 (1.9–2.5)	1.2 (0.9–1.7)	0.302
Isolated	1.6 (1.1–2.4)	1.6 (1.3–2.0)	1.0 (0.6–1.5)	0.960
**Federal poverty level^§§^**				
≥400%	22.9 (19.8–26.3)	29.8 (28.2–31.5)	0.8 (0.7–0.9)	0.001^§^
200%–399%	27.0 (22.8–31.7)	28.7 (27.0–30.4)	0.9 (0.8–1.1)	0.488
100%–199%	24.2 (20.4–28.4)	22.3 (20.5–24.2)	1.1 (0.9–1.3)	0.409
<100%	25.9 (22.1–30.0)	19.2 (17.4–21.1)	1.4 (1.1–1.6)	0.002^§^
**Health care**				
Inadequate or no insurance^¶¶^	33.8 (30.2–37.7)	25.4 (23.9–27.1)	1.3 (1.2–1.5)	<0.001^§^
Public insurance***	51.1 (47.2–54.9)	34.4 (32.5–36.3)	1.5 (1.4–1.6)	<0.001^§^
Lacks a medical home^†††^	58.1 (54.3–61.8)	48.2 (46.3–50.0)	1.2 (1.1–1.3)	<0.001^§^
Child saw health care provider in past year^§§§^	90.0 (86.3–92.7)	87.6 (86.1–88.9)	1.0 (1.0–1.1)	0.174
Needed care not received^¶¶¶^	7.0 (5.1–9.4)	1.7 (1.1–2.5)	4.2 (2.5–6.9)	<0.001^§^
**Family**				
Fair or poor parental mental health****	13.7 (10.9–17.1)	5.7 (4.9–6.7)	2.4 (1.8–3.2)	<0.001^§^
Fair or poor parental physical health^††††^	15.7 (12.8–19.2)	8.1 (7.0–9.2)	2.0 (1.5–2.5)	<0.001^§^
Difficult to get by on family's income^§§§§^	38.0 (34.2–42.0)	21.3 (19.7–22.9)	1.8 (1.6–2.0)	<0.001^§^
Parent lacks emotional support^¶¶¶¶^	21.2 (17.9–24.9)	23.3 (21.4–25.3)	0.9 (0.8–1.1)	0.299
Child care problems (ages 0–5 only)*****	18.8 (13.8–25.2)	5.3 (4.4–6.3)	3.5 (2.5–5.0)	<0.001^§^
**Community**				
Neighborhood without amenities^†††††^	65.2 (61.3–68.9)	60.3 (58.5–62.0)	1.1 (1.0–1.2)	0.023^§^
Neighborhood in poor condition^§§§§§^	26.8 (23.4–30.6)	24.5 (22.8–26.2)	1.1 (0.9–1.3)	0.245
Lack of support in neighborhood^¶¶¶¶¶^	35.7 (31.7–39.9)	26.5 (24.7–28.4)	1.3 (1.2–1.5)	<0.001^§^
Neighborhood perceived to lack safety******	6.8 (4.8–9.5)	5.4 (4.4–6.6)	1.3 (0.8–1.9)	0.300

**Abbreviation:** CI = confidence interval.

* Based on a response of “yes” to whether “a doctor or other health care provider ever told you that this child has” one or more of the following disorders: “anxiety problems, depression, attention-deficit/hyperactivity disorder, behavioral or conduct problems, Tourette syndrome, autism spectrum disorder, learning disability, intellectual disability, developmental delay, or speech or other language disorder.”

^†^ Percentages are weighted. Column percentages might not sum to 100% because of rounding.

^§^ p-value for weighted Wald chi-square test. All p-values <0.05 indicate statistically significant differences from “No MBDD.”

^¶^ Missing data on sex were imputed for 0.1% of the sample using hot-deck imputation methods.

** Missing data on race and ethnicity were imputed for 0.3% and 0.5% of the sample, respectively, using hot-deck imputation methods. “Other, non-Hispanic” includes American Indian/Alaska Native, Native Hawaiian or Other Pacific Islander, and Asian.

^††^ Urban and rural designations were determined using a four-category classification based on 2010 rural-urban community area codes (RUCAs), a census tract–based classification system. Urban areas (RUCA codes 1.0, 1.1, 2.0, 2.1, 3.0, 4.1, 5.1, 7.1, 8.1, and 10.1) include metropolitan areas and surrounding towns from which commuters flow to an urban area; large rural areas (RUCA codes 4.0, 5.0, and 6.0) include large towns (micropolitan areas) with populations of 10,000–49,999 and their surrounding areas; small rural areas (RUCA codes 7.0, 7.2, 8.0, 8.2, and 9.0) include small towns with populations of 2,550–9,999 and up to 50% secondary flow to a large urban cluster of up to 50,000; and isolated areas (RUCA codes 10.0, 10.2, and 10.3) with less than 2,500 population and up to 50% secondary flow to a large or small urban cluster (population up to 10,000). (https://www.census.gov/geo/reference/ua/urban-rural-2010.html).

^§§^ Federal poverty level is based on family income and family size and composition using federal poverty thresholds that are updated annually by the U.S. Census Bureau using the change in the average annual consumer price index for all urban consumers. Imputed income was used for 16.6% of children aged 2–8 years with MBDD status and sex reported, but without reported household income, using regression methods.

^¶¶^ Based on a negative value for any of four variables based on these questions: 1) “Is this child currently covered by any kind of health insurance or health coverage plan?” 2) “How often does this child’s health insurance offer benefits or cover services that meet this child’s needs?” 3) “Does the family pay out-of-pocket expenses,” and if yes, ”How often are these costs reasonable?” and 4) “How often does this child’s health insurance allow him or her to see the health care providers he or she needs?”

*** Based on a response of “yes” to having “Medicaid, Medical Assistance, or any kind of government assistance plan for those with low incomes or a disability.”

^†††^ Based on five component variables (personal doctor or nurse, usual source for sick and well care, family-centered care, problems getting needed referrals, satisfaction with communication, and effective care coordination when needed), derived from 16 survey items. To have a medical home, the child must have a personal doctor or nurse, usual source of care, and family-centered care; children needing referrals or care coordination must also have those criteria met.

^§§§^ Whether the child saw a health care provider in the last 12 months was based on a response of “yes” to the following question: “During the past 12 months, did this child see a doctor, nurse, or other health care professional for sick-child care, well-child check-ups, physical exams, hospitalizations, or any other kind of medical care?”

^¶¶¶^ Based on a response of “yes” to the following question: “During the past 12 months, was there any time when this child needed health care, but it was not received? By health care, we mean medical care as well as other kinds of care like dental care, vision care, and mental health services.”

**** Based on whether either parent reported “fair” or “poor” (i.e., compared with “excellent,” “very good,” or “good”) to the question “In general, how is your mental or emotional health?”

^††††^ Based on whether either parent reported “fair” or “poor” (i.e., compared with “excellent,” “very good,” or “good”) to the question: “In general, how is your physical health?”

^§§§§^ Based on an answer of “very often” or “somewhat often” (i.e., compared with “never” or “rarely”) to the question: “Since this child was born, how often has it been very hard to get by on your family's income (e.g., hard to cover the basics like food or housing)?”

^¶¶¶¶^ Based on a response of “no” to the question “During the past 12 months, was there someone that you could turn to for day-to-day emotional support with parenting or raising children?”

***** Based on a response of "yes" to the question: "During the past 12 months, did you or anyone in the family have to quit a job, not take a job, or greatly change your job because of problems with child care for (child)?". Note: This question was asked for children aged 0-5 years only.

^†††††^ Based on a response of “no” to any of the following four questions: “In your neighborhood, is/are there: 1) sidewalks or walking paths?; 2) a park or playground?; 3) a recreation center, community center, or boys’ and girls’ club?; 4) a library or bookmobile?”

^§§§§§^ Based on a response of “yes” to any of the following three questions: “In your neighborhood, is/are there: 1) litter or garbage on the street or sidewalk?; 2) poorly kept or rundown housing?; 3) vandalism such as broken windows or graffiti?”

^¶¶¶¶¶^ Based on a response of “definitely disagree” or “somewhat disagree” (i.e., compared with “definitely agree” or “somewhat agree”) to any of the following three questions: “To what extent do you agree with these statements about your neighborhood or community? 1) People in this neighborhood help each other out; 2) We watch out for each other’s children in this neighborhood; 3) When we encounter difficulties, we know where to go for help in our community.”

****** Based on a response of “definitely disagree” or “somewhat disagree” (i.e., compared with “definitely agree” or “somewhat agree”) to the following statement: “This child is safe in our neighborhood.”

Prevalence of MBDDs was higher in each consecutive decreasing income level compared with the highest level (≥400% of FPL) (Table 2); estimates of MBDDs ranged from 13.9% among those in the highest income level (≥400% of FPL) to 22.1% among those in the lowest level (<100% of FPL). A lower percentage of children in lower-income households saw a health care provider in the past 12 months (80.4%) and a higher percentage did not receive needed care (5%), compared with children in the highest income level (93.8% and 0.8%, respectively). Similar patterns across income levels were found for most health care, family, and community factors (e.g., increasing prevalences of the risk factors as household income level decreased), with the exception that inadequate insurance was less often reported for children in the lower income levels than for those in the highest level.

**TABLE 2:** Prevalence of parental report of any mental, behavioral, or developmental disorder (MBDD), and health care, family, and community factors among children aged 2–8 years, by federal poverty level — National Survey of Children’s Health, United States, 2016

Characteristic	Percentage of federal poverty level*							
	≥400% (referent)	200%–399%		100%–199%		<100%		Overall
	% (95% CI)^†^	% (95% CI)^†^	PR (95% CI)	% (95% CI)^†^	PR (95% CI)	% (95% CI)^†^	PR (95% CI)	% (95% CI)^†^
**MBDD^§^**	13.9 (12.1–16.0)	16.6 (14.1–19.3)	1.2 (0.9–1.5)	18.6 (15.5–22.1)	1.3 (1.1–1.7)^¶^	22.1 (18.8–25.9)	1.6 (1.3–2.0)^¶^	17.4 (16.2–18.7)
**Health care**								
Inadequate or no insurance**	27.4 (25.2–29.7)	33.0 (30.2–36.0)	1.2 (1.1–1.4)^¶^	24.1 (20.5–28.0)	0.9 (0.7–1.0)	20.7 (16.9–25.2)	0.8 (0.6–0.9)^¶^	26.9 (25.5–28.4)
Public insurance^††^	6.6 (4.7–9.2)	21.8 (19.0–24.8)	3.3 (2.2–5.0)^¶^	61.6 (57.6–65.4)	9.4 (6.7–13.2)^¶^	76.3 (71.6–80.5)	11.7 (8.2–16.6)^¶^	37.3 (35.5–39.0)
Lacks a medical home^§§^	36.7 (34.4–39.0)	48.2 (45.2–51.3)	1.3 (1.2–1.4)^¶^	57.7 (53.7–61.7)	1.6 (1.4–1.7)^¶^	62.1 (57.7–66.4)	1.7 (1.5–1.9)^¶^	49.9 (48.2–51.5)
Child saw health care provider in past year^¶¶^	93.8 (92.4–95.0)	90.1 (88.0–91.8)	1.0 (0.9–1.0)^¶^	84.7 (80.8–88.0)	0.9 (0.9–0.9)^¶^	80.4 (75.6–84.5)	0.9 (0.8–0.9)^¶^	88.0 (86.6–89.2)
Needed care not received***	0.8 (0.5–1.2)	1.9 (1.3–2.7)	2.4 (1.4–4.4)^¶^	3.6 (2.3–5.6)	4.6 (2.4–9.1)^¶,†††^	5.0 (3.0–8.2)	6.4 (3.2–12.6)^¶,†††^	2.6 (2.0–3.3)
**Family**								
Fair or poor parental mental health^§§§^	3.9 (2.8–5.5)	6.1 (4.3–8.6)	1.6 (1.0–2.6)	10.5 (7.9–13.7)	2.7 (1.7–4.2)^¶^	15.4 (12.2–19.1)	3.9 (2.6–5.8)^¶^	8.0 (7.0–9.1)
Fair or poor parental physical health^¶¶¶^	3.4 (2.4–4.7)	8.5 (6.5–11.1)	2.6 (1.7–3.9)^¶^	14.6 (11.5–18.4)	4.4 (2.9–6.7)^¶^	21.9 (18.1–26.2)	6.6 (4.5–9.6)^¶^	10.6 (9.4–11.8)
Difficult to get by on family's income****	6.1 (4.8–7.7)	19.9 (17.3–22.8)	3.3 (2.4–4.5)^¶^	34.6 (30.7–38.8)	5.7 (4.3–7.5)^¶^	45.0 (40.2–50.0)	7.4 (5.8–9.4)^¶^	24.2 (22.7–25.7)
Parent lacks emotional support^††††^	13.0 (11.1–15.0)	18.2 (15.5–21.2)	1.4 (1.1–1.8)^¶^	29.2 (24.9–34.0)	2.3 (1.8–2.8)^¶^	36.9 (32.0–42.1)	2.9 (2.3–3.5)^¶^	22.9 (21.2–24.7)
Child care problems (ages 0–5 yrs only)^§§§§^	3.4 (2.4–4.6)	8.0 (5.7–10.9)	2.4 (1.5–3.7)^¶^	7.8 (5.4–11.1)	2.3 (1.4–3.8)^¶^	10.7 (7.9–14.4)	3.2 (2.0–5.0)^¶^	7.1 (6.0–8.3)
**Community**								
Neighborhood without amenities^¶¶¶¶^	51.3 (49.0–53.6)	61.6 (58.7–64.3)	1.2 (1.1–1.3)^¶^	65.6 (61.0–69.9)	1.3 (1.2–1.4)^¶^	70.1 (65.1–74.7)	1.4 (1.3–1.5)^¶^	61.1 (59.5–62.7)
Neighborhood in poor condition*****	15.0 (13.3–16.9)	23.2 (20.5–26.0)	1.5 (1.3–1.8)^¶^	28.4 (24.5–32.7)	1.9 (1.6–2.3)^¶^	38.1 (33.4–42.9)	2.5 (2.1–3.0)^¶^	24.9 (23.4–26.4)
Lack of support in neighborhood^†††††^	15.5 (13.6–17.5)	25.7 (22.4–29.2)	1.7 (1.4–2.0)^¶^	35.0 (30.7–39.6)	2.3 (1.9–2.7)^¶^	41.8 (37.0–46.8)	2.7 (2.3–3.2)^¶^	28.0 (26.4–29.7)
Neighborhood perceived to lack safety^§§§§§^	1.5 (0.9–2.6)	4.6 (3.4–6.3)	3.0 (1.8–5.2)^¶^	6.7 (4.6–9.8)	4.4 (2.4–8.2)^¶,†††^	11.9 (8.6–16.4)	7.9 (4.4–14.2)^¶^	5.6 (4.7–6.7)
Urban/Rural status^¶¶¶¶¶^								
Urban	94.6 (93.8–95.3)	90.2 (89.1–91.2)	1.0 (0.9–1.0)^¶^	89.4 (87.8–90.9)	0.9 (0.9–1.0)^¶^	87.9 (85.5–90.0)	0.9 (0.9–1.0)^¶^	90.8 (90.1–91.5)
Large rural	3.4 (2.8–4.1)	5.6 (4.9–6.4)	1.6 (1.3–2.1)^¶^	6.1 (5.0–7.5)	1.8 (1.4–2.4)^¶^	6.6 (5.1–8.5)	1.9 (1.4–2.7)^¶^	5.3 (4.8–5.8)
Small rural	1.3 (1.0–1.7)	2.3 (1.8–2.8)	1.7 (1.2–2.5)^¶^	2.3 (1.8–3.0)	1.8 (1.2–2.6)^¶^	3.4 (2.5–4.6)	2.6 (1.7–3.9)^¶^	2.2 (2.0–2.6)
Isolated	0.6 (0.5–0.9)	2.0 (1.5–2.5)	3.0 (2.1–4.5)^¶^	2.1 (1.5–2.8)	3.2 (2.1–5.0)^¶^	2.1 (1.3–3.3)	3.2 (1.9–5.7)^¶^	1.6 (1.4–1.9)

**Abbreviations:** CI = confidence interval; PR = prevalence ratio.

* Federal poverty level is based on family income and family size and composition using federal poverty thresholds that are updated annually by the U.S. Census Bureau using the change in the average annual consumer price index for all urban consumers. Imputed income was used for 16.6% of children aged 2–8 years with MBDD status and sex reported, but without reported household income, using regression methods.

^†^ Percentages are weighted. Column percentages might not sum to 100% because of rounding.

^§^ Based on a response of “yes” to whether “a doctor or other health care provider ever told you that this child has” one or more of the following disorders: “anxiety problems, depression, attention-deficit/hyperactivity disorder, behavioral or conduct problems, Tourette syndrome, autism spectrum disorder, learning disability, intellectual disability, developmental delay, or speech or other language disorder.”

^¶^ Statistically significant difference from the referent group.

** Based on a negative value for any of four variables based on these questions: 1) “Is this child currently covered by any kind of health insurance or health coverage plan?” 2) “How often does this child’s health insurance offer benefits or cover services that meet this child’s needs?” 3) “Does the family pays out-of-pocket expenses,” and if yes, “How often are these costs reasonable?” and 4) “How often does this child’s health insurance allow him or her to see the health care providers he or she needs?”

^††^ Based on a response of “yes” to having “Medicaid, Medical Assistance, or any kind of government assistance plan for those with low incomes or a disability.”

^§§^ Based on five component variables (personal doctor or nurse, usual source for sick and well care, family-centered care, problems getting needed referrals, satisfaction with communication, and effective care coordination when needed), derived from 16 survey items. To have a medical home, the child must have a personal doctor or nurse, usual source of care, and family-centered care; children needing referrals or care coordination must also have those criteria met.

^¶¶^ Based on a response of “yes” to the following question: “During the past 12 months, did this child see a doctor, nurse, or other health care professional for sick-child care, well-child check-ups, physical exams, hospitalizations or any other kind of medical care?”

*** Based on a response of “yes” to the following question: “During the past 12 months, was there any time when this child needed health care but it was not received? By health care, we mean medical care as well as other kinds of care like dental care, vision care, and mental health services.”

^†††^ Estimate has a relative standard error >30% and might be unreliable.

^§§§^ Based on whether either parent reported “fair” or “poor” (i.e., compared with “excellent,” “very good,” or “good”) to the question: “In general, how is your mental or emotional health?”

^¶¶¶^ Based on whether either parent reported “fair” or “poor” (i.e., compared with “excellent,” “very good,” or “good”) to the question “In general, how is your physical health?”

**** Based on an answer of “very often” or “somewhat often” (i.e., compared with “never” or “rarely”) to the question “Since this child was born, how often has it been very hard to get by on your family's income (hard to cover the basics like food or housing)?”

^††††^ Based on a response of “yes” to the question “During the past 12 months, was there someone that you could turn to for day-to-day emotional support with parenting or raising children?”

^§§§§^ Based on a response of “yes” to the question: “During the past 12 months, did you or anyone in the family have to quit a job, not take a job, or greatly change your job because of problems with child care for (child)? Note: This question was asked for children aged 0–5 years only.

^¶¶¶¶^ Based on a response of “no” to any of the following four questions: “In your neighborhood, is/are there: 1) sidewalks or walking paths? 2) a park or playground? 3) a recreation center, community center, or boys’ and girls’ club? 4) a library or bookmobile?”

***** Based on a response of “yes” to any of the following three questions: “In your neighborhood, is/are there: 1) Litter or garbage on the street or sidewalk? 2) Poorly kept or rundown housing? 3) Vandalism such as broken windows or graffiti?”

^†††††^ Based on a response of “definitely disagree” or “somewhat disagree” (i.e., compared with “definitely agree” or “somewhat agree”) to any of the following three questions: “To what extent do you agree with these statements about your neighborhood or community? 1) People in this neighborhood help each other out, 2) We watch out for each other’s children in this neighborhood, 3) When we encounter difficulties, we know where to go for help in our community.”

^§§§§§^ Based on a response of “definitely disagree” or “somewhat disagree” (i.e., compared with “definitely agree” or “somewhat agree”) to the following question: “To what extent do you agree with these statements about your neighborhood or community? 1) This child is safe in our neighborhood.”

^¶¶¶¶¶^ Urban and rural designations were determined using a four-category classification based on 2010 rural-urban community area codes (RUCAs), a census tract–based classification system. Urban areas (RUCA codes 1.0, 1.1, 2.0, 2.1, 3.0, 4.1, 5.1, 7.1, 8.1, and 10.1) include metropolitan areas and surrounding towns from which commuters flow to an urban area; large rural areas (RUCA codes 4.0, 5.0, and 6.0) include large towns (micropolitan areas) with populations of 10,000–49,999 and their surrounding areas; small rural areas (RUCA codes 7.0, 7.2, 8.0, 8.2, and 9.0) include small towns with populations of 2,550–9,999 and up to 50% secondary flow to a large urban cluster of up to 50,000; isolated areas (RUCA codes 10.0, 10.2, and 10.3) with less than 2,500 population and up to 50% secondary flow to a large or small urban cluster (population up to 10,000). (https://www.census.gov/geo/reference/ua/urban-rural-2010.html).

Among children living at <200% of FPL, 82.6% saw a health care provider in the past year, and 73.4% received public assistance (Table 3). Among the children who did not see a health care provider in the past year, 69.0% received public assistance and 19.2% had a diagnosed MBDD. Among children who did not see a health care provider in the past year and had a diagnosed MBDD, 81.7% received public assistance. Of children who did not see a health care provider in the past year and did not have a diagnosed MBDD, 66.0% received public assistance.

**TABLE 3:** Service use among children* living below 200% of the federal poverty level, by parental report of any mental, behavioral, and developmental disorder (MBDD) — National Survey of Children’s Health, United States, 2016

Characteristic	No public assistance^†^	Public assistance^†^	Total
	% (95% CI)^§^	% (95% CI)^§^	% (95% CI)^§^
**Child saw health care provider in the past year^¶^**	25.7 (23.1–28.4)	74.3 (71.6–76.9)	82.6 (79.7–85.2)
With MBDD**	15.1 (11.6–19.6)	84.9 (80.4–88.4)	21.1 (18.5–24.0)
Without MBDD**	28.5 (25.5–31.7)	71.5 (68.3–74.5)	78.9 (76.0–81.5)
**Child did not see health care provider in the past year^¶^**	31.1 (24.2–38.7)	69.0 (61.3–75.8)	17.4 (14.8–20.3)
With MBDD**	18.3^††^ (9.1–33.3)	81.7 (66.7–90.9)	19.2 (13.0–27.5)
Without MBDD**	34.0 (26.1–42.9)	66.0 (57.1–73.9)	80.8 (72.5–87.0)
**Total**	26.6 (24.1–29.2)	73.4 (70.8–75.9)	—

**Abbreviation:** CI = confidence interval.

* Restricted to nonmissing responses for child MBDD status, whether the child’s family received public assistance, and whether the child saw a health care provider in the past year.

^†^ Based on whether the parent reported the family received any of the four benefits (cash assistance; Women, Infants, and Children; Supplemental Nutrition Assistance Program; or free or reduced cost meals at school) at any time during the past 12 months.

^§^ Percentages are weighted. Column and row percentages might not sum to 100% because of rounding.

^¶^ Based on response to the following question: “During the past 12 months, did (child) see a doctor, nurse, or other health care professional for sick-child care, well-child check-ups, physical exams, hospitalizations, or any other kind of medical care?”

** Based on response to whether “a doctor or other health care provider ever told you that this child has” one or more of the following disorders: “anxiety problems, depression, attention-deficit/hyperactivity disorder, behavioral or conduct problems, Tourette syndrome, autism spectrum disorder, learning disability, intellectual disability, developmental delay, or speech or other language disorder.”

^††^ Estimate is unstable; relative standard error = 33.3%.

## Discussion

Consistent with previous studies (*3*,*5*,*7*), this study found that children living in lower-income households had higher prevalences of a parent-reported diagnosis of an MBDD and other health care, family, and community risk factors associated with MBDDs than did children living in higher-income households. Most children had seen a health care provider in the past year regardless of income level; therefore, the American Academy of Pediatrics recommendation to screen for MBDDs (*8*) and family and socioeconomic risk factors (*4*) during primary care visits appears to be theoretically feasible.

Screening**^,††^ in health care settings can be challenging in practice, and MBDDs might be underdiagnosed even among children who have recently seen a health care provider (*9*). Children living in lower-income households had lower prevalences of having seen a health care provider in the past year and of receiving needed health care compared with children living in higher-income households. Approximately one in five children living at <200% of FPL who did not see a health care provider in the past year had a diagnosed MBDD. This, coupled with families with lower incomes reporting greater difficulty receiving needed health care, raises concern that MBDDs might be undertreated in this population. Additionally, families living in poverty were more likely to experience a range of risk factors related to MBDDs; therefore, connections to health care services are especially relevant for this population.

Public assistance programs might provide opportunities to connect families living in poverty to services, in line with the American Academy of Pediatrics call for collaboration between public health professionals and pediatricians (*10*). Where treatment resources are available, education or early identification programs could be embedded within services families are already accessing. For example, CDC’s Learn the Signs. Act Early program connects WIC staff members with resources for parents about early identification of developmental delays and helps staff with referrals to primary care.^§§^ Similar approaches to promoting parental awareness of MBDDs and the value of pediatric screening, if carefully designed to minimize stigmatization, could be implemented within other public assistance programs. Identification of MBDDs and associated risk factors (e.g., poor parental mental health or lack of support) and connection to services can be challenging for families, even among those with primary care. Therefore, expanded co-location of developmental and behavioral health services in public assistance programs, as well as other sites that would reach additional families (e.g., schools or early-learning settings, federally qualified health centers,^¶¶^ or federal partnerships***), might help to eliminate barriers to care for families living in poverty.^†††^^,^^ §§§^

The findings in this report are subject to at least three limitations. First, data are cross-sectional, so it was not possible to ascertain temporal associations or causality. Second, the sampling weights used to calculate nationally representative estimates might not completely compensate for nonresponse bias. Finally, indicators rely on parental report and might be subject to recall or social desirability bias.

Early identification and treatment of MBDDs could positively impact a child’s functioning and reduce the need for costly interventions over time (*8*). Public assistance programs hold potential for increasing developmental monitoring and connection to treatment for MBDDs for families living in poverty by collaborating to distribute resources, implementing co-located screening services, or facilitating connections to appropriate treatment and care.

**Summary**

**What is already known about this topic?**

Poverty, as well as health care, family, and community factors are associated with mental, behavioral, and developmental disorders (MBDDs) in children.

**What is added by this report?**

Parent-reported data from 2016 showed that a higher percentage of children in lower-income households had ever received a diagnosis of an MBDD and a lower percentage had seen a health care provider in the previous year, compared with children in higher-income households. Most children in lower-income households were in families receiving public assistance benefits.

**What are the implications for public health practice?**

Public assistance programs might offer collaboration opportunities for public health and pediatrics to provide information, implement co-located screening programs or services, or facilitate connection to care.

## Footnotes

*
https://mchb.hrsa.gov/data/national-surveys/data-user .

† https://census.gov/content/dam/Census/programs-surveys/nsch/tech-documentation/nonresponse-bias-analysis/NSCH%202016%20Nonresponse%20Bias%20Analysis.pdf.

§
https://www.census.gov/programs-surveys/nsch/technical-documentation/codebooks.html .

¶
https://census.gov/content/dam/Census/programs-surveys/nsch/tech-documentation/methodology/2016-NSCH-Methodology-Report.pdf .

**
https://www.aap.org/en-us/advocacy-and-policy/aap-health-initiatives/Screening/Pages/default.aspx .

§§
https://www.cdc.gov/ncbddd/actearly/wic-providers.html .

¶¶
https://www.hrsa.gov/opa/eligibility-and-registration/health-centers/fqhc/index.html .

***
https://healthysafechildren.org/grantee/project-launch .

†††
https://www.milbank.org/publications/behavioral-health-integration-in-pediatric-primary-care-considerations-and-opportunities-for-policymakers-planners-and-providers/ .

§§§ https://www2.ed.gov/about/inits/ed/earlylearning/files/health-early-learning-statement.pdf.
